# Needs assessment: towards a more responsive Canadian Society of Nephrology Annual General Meeting (CSN AGM) program

**DOI:** 10.1186/s40697-016-0121-x

**Published:** 2016-06-24

**Authors:** Barry A. Cohen, Mark J. Courtney, Louise M. Moist, James Barton

**Affiliations:** Section of Nephrology, Department of Medicine, University of Manitoba, Winnipeg, MB Canada; Division of Nephrology and Immunology, Department of Medicine, University of Alberta, 11-107 Clinical Sciences Building, 11350 83 Ave., Edmonton, AB Canada; Department of Medicine, Schulich School of Medicine, Kidney Clinical Research Unit, London Health Sciences Centre, Western University, 800 Commissioners Rd., Rm A2-38, London, ON N6A-5W9 Canada; Division of Nephrology, Department of Medicine, Saskatoon Nephrology Group, University of Saskatchewan, 434-230 Avenue R South, Saskatoon, SK S7M-2Z1 Canada

**Keywords:** Needs assessment, Perceived needs, Unperceived needs, Environmental scan, Questionnaire, Continuing medical education, Nephrology

## Abstract

**Background:**

A critical feature of any continuing medical education (CME) program is the inclusion of a needs assessment for its target audience. This assessment must identify both perceived and unperceived needs, so as to best capture the entire spectrum of learning opportunities for the group.

**Objective:**

We describe the process developed by the Canadian Society of Nephrology (CSN) to enhance the educational effectiveness of its Annual General Meeting program.

**Design:**

The design of this study is the analysis of a survey questionnaire and of the Canadian Organ Replacement Registry (CORR) database.

**Participants:**

We surveyed members of the CSN and analyzed patient data from CORR aggregated by center.

**Measurements:**

We tabulated votes in the survey by topic. We assessed the extent to which centers achieved CSN guideline targets on the clinical management of patients on dialysis.

**Methods:**

Perceived needs: a CSN panel constructed a list of topics, which was amplified by the inclusion of topics based on members’ text responses to open-ended questions during previous iterations of this process. CSN members specified their top five choices, using an online survey instrument. Unperceived needs: an expert panel determined achievable thresholds for a number of quality metrics associated with dialysis. The quality metrics were identified from CSN guidelines. Using patient data in the CORR database, we generated center-specific performance estimates for each quality metric and constructed ratios comparing the performance of each center with the achievable threshold. We triangulated the results of the two assessments.

**Results:**

The response rate for the perceived needs assessment survey was 16 %. This assessment identified “Primary and Secondary Glomerulonephritis” as the non-dialysis topics and “Infectious Complications of Dialysis Access” and “Volume Status and Hypertension on Dialysis” as the dialysis topics with the highest perceived learning needs. In the unperceived needs assessment, “Vascular Access Type” and “Vascular Access Monitoring” were identified as having the highest learning needs. Triangulation identified “Vascular Access Type” and “Vascular Access Monitoring” as high needs topics.

**Limitations:**

Perceived needs assessment: Some topics were much more general than others, which could have led to over-selection. The response rate of 16 % limits the robustness of generalization to the membership as a whole or to all meeting attendees.

Unperceived needs assessment: The assessment was limited by the data that CORR actually collects; many aspects of general nephrology practice, including glomerulonephritis, are not covered. The level of evidence underlying the various guidelines was variable, and in some cases, poor. A validated approach to data analysis in this area is lacking.

**Conclusions:**

To our knowledge, this is the first published example of a needs assessment for a nephrology CME event that considers both the perceived and unperceived needs of the membership. The results of this exercise are currently being used to assist in the development of a more responsive CME program.

## Abrégé

**Contexte:**

Un aspect crucial de tout programme d’enseignement médical continu (EMC) est l’inclusion d’une évaluation des besoins de la clientèle cible. Cet examen se doit de permettre l’identification tant des besoins perçus que des besoins non perçus afin de mieux cerner les occasions d’apprentissage pour le groupe.

**Objectifs de l’étude:**

Cet article fait la description de la démarche mise au point par la Société de Néphrologie du Canada (SNC) dans le cadre du programme de son assemblée générale annuelle. Cette démarche vise accroître l’efficacité de la formation.

**Plan de l’étude:**

On a procédé à l’analyse des réponses obtenues lors d’une enquête auprès des membres ainsi qu’à la consultation des métadonnées du Registre Canadien des Insuffisances et des Transplantations d’Organes (RCITO).

**Participants:**

Le sondage a été mené auprès des membres de la SCN et on a procédé à l’examen des données du RCITO cumulées dans chaque centre de soins.

**Mesures:**

La compilation des réponses au sondage a été effectuée par catégories. Nous avons également évalué dans quelle mesure avaient été atteints les objectifs d’orientation fixés par les lignes directrices de la SCN quant à la conduite du traitement clinique des patients sous dialyse.

**Méthodologie:**

Un premier volet s’attardait à définir les besoins perçus. Pour ce faire, le comité de la SCN a dressé une liste de thèmes. Cette liste s’est par la suite allongée avec l’ajout de sujets tirés des réponses des membres aux questions ouvertes colligées lors des versions antérieures du sondage. Les membres de la SCN ont spécifié les cinq thèmes qu’ils jugeaient les plus importants par le biais d’un instrument de sondage en ligne. Un second volet se penchait sur les besoins non perçus. Dans ce deuxième cas, un groupe d’experts a déterminé les seuils réalisables pour un certain nombre de paramètres de la qualité associés à la dialyse. L’identification de ces paramètres était tirée des lignes directrices de la SCN. À l’aide des données des patients contenues dans le RCITO, une appréciation de la performance de chaque centre de soins a été produite pour chacun des paramètres identifiés. Des ratios ont ensuite été construits en comparant la performance de chaque centre de soins avec le seuil réalisable établi précédemment. De plus, les résultats des deux analyses ont été triangulés.

**Résultats:**

Le taux de réponse pour le sondage sur l’analyse des besoins perçus a été de 16 %. Cet examen a permis d’identifier les besoins perçus à considérer en matière de formation pour les futurs néphrologues. Du côté des sujets non reliés directement à la dialyse elle-même, on retrouve la glomérulonéphrite primaire et secondaire ainsi que les complications infectieuses liées au cathéter. Quant aux sujets directement reliés à la dialyse, on a pu identifier la volémie et l’hypertension en dialyse. En ce qui concerne les besoins non perçus, le type d’accès vasculaire et la surveillance de celui-ci ont été identifiés comme étant des sujets important à couvrir lors de la formation. Ces deux derniers sujets sont également ressortis comme très importants à considérer dans les besoins de formation lors de la triangulation des résultats.

**Limites de l’étude:**

Dans le cas des besoins perçus, certains sujets étaient de nature très générale et ceci pourrait avoir conduit à une sélection excessive. Qui plus est, le très faible taux de réponse limite la robustesse d’une généralisation des résultats à tous les membres ayant participé à l’assemblée. Du côté des besoins non perçus, l’analyse est limitée par les données du RCITO où plusieurs aspects de la pratique générale en néphrologie, notamment la prise en charge de la glomérulonéphrite, ne sont pas couverts. Le degré d’éléments probants sous-jacent les différents principes directeurs s’est avéré variable et dans certains cas, médiocre. Une approche validée face à l’analyse des données dans ce domaine est manquante.

**Conclusions:**

À notre connaissance, cette enquête constitue la première analyse publiée des besoins, réalisée lors d’un événement d’EMC en néphrologie, qui prend en considération les besoins perçus et non perçus de ses membres. Les résultats de cet exercice sont actuellement utilisés dans le développement d’un programme d’EMC mieux adapté.

## What was known before

A needs assessment of the target audience improves the effectiveness of any continuing medical education program. This should include an assessment of both perceived and unperceived needs. For this reason, the Royal College of Physicians and Surgeons of Canada (RCPSC) has made the inclusion of such an assessment a requirement for accreditation.

## What this adds

A needs assessment that considers both perceived and unperceived needs can be conducted for the Canadian Society of Nephrology in order to enhance the effectiveness of its Annual General Meeting program. This assessment has identified several specific topics that should be discussed at this meeting.

## Background

The primary purpose of continuing medical education (CME) is to enhance physicians’ knowledge and behavior in their respective fields, in order to improve patient health outcomes. As such, improvements in health outcomes, not increases in physician knowledge, are the ultimate goals of any CME program. A number of factors have been shown to improve the effectiveness of a CME program, including interactive programming (as opposed to didactic), inclusion of enabling, knowledge translation factors (e.g., patient educational materials), and longitudinal programming; additionally, that programming is based on a needs assessment [1–3].

Indeed, the importance of a needs assessment to CME has been known for more than a generation. In 1977, Bertram and Brooks-Bertram stated that “the gross failure to demonstrate effectiveness of CME is chiefly due to failure to identify the learning needs of practitioners and the health needs of their patients as well as inadequate evaluation methods” [4]. This claim has been echoed by multiple authors [1–3].

A needs assessment can be defined as a systematic process designed to collect and analyze information as to the specific learning needs of a particular group; the activity must therefore focus on this target group. A learning need may be defined as the measured gap between an individual’s (or group’s) current knowledge as compared to either (i) an agreed-upon level of knowledge (optimal knowledge) or importance of such knowledge or (ii) a gap between an accepted standard of practice and an individual’s current level of practice. Perceived needs are the self-identified learning needs of the target group (i.e., the “wants” of the group). They are subjectively determined and are best exemplified by the phrase, “I know what I don’t know.” Unperceived needs represent discrepancies not perceived by the learners; they are objectively determined and are best exemplified by the phrase, “What I don’t know that I don’t know” [1, 5].

In 2009, the Royal College of Physicians and Surgeons of Canada (RCPSC) instructed the Canadian Society of Nephrology (CSN) to conduct a learning needs assessment of the CSN membership, as part of the development of the CSN Annual General Meeting (AGM), in order to meet the accreditation requirement, as per Standard 5 of the RCPSC Accreditation Guide for Continuing Professional Development (CPD) Events for Specialists. This standard further states that there must be a balance between perceived and unperceived needs, which should include strategies to identify practice standards [6].

The Education Committee of the CSN (EC-CSN) subsequently developed the required needs assessment tool during 2010 and 2011 and first utilized it in the planning of the 2012 AGM. What follows is a description of the development of the needs assessment tool, as well as the results of its most recent application.

## Methods

We judged that this needs assessment did not require research ethics board review and considered participation in the survey to imply consent.

(A) Perceived needs

In the spring of 2010, a survey questionnaire was initially developed by the EC-CSN to determine which subjects (in the areas of general nephrology and dialysis) were prioritized by CSN members and other conference attendees (e.g., former and retired members, general internists who had attended previous meetings) as to their own learning needs. The topic list was first developed by looking at the topics listed on the recently developed Syllabus of Training Objectives for Nephrology Trainees—Medical Expert Role [7]. A roundtable discussion ensued, and the final topic list was developed. At every subsequent annual EC-CSN meeting, the topic list was updated, based on the responses from the previous year. For example, if one topic was listed as a “write-in” topic by multiple respondents, then this topic appeared as a choice on subsequent years’ surveys. The survey described in this paper was conducted in the spring of 2015. Potential conference attendees listed their top five topics for future AGM presentations using an online tool (surveymonkey.com). The results were tabulated, and the topics were ranked in order of membership preference. In addition, those topics specifically related to dialysis were ranked separately. Finally, topics that fell within the highest tertile of votes received were considered “high needs topics,” those that fell within the middle tertile of votes received were considered “intermediate needs topics,” and those that fell within the lowest tertile of votes received were considered “low needs topics.”

(B) Unperceived needs

Assessment of the target group’s unperceived needs was facilitated using an environmental scan—the Canadian Organ Replacement Registry (CORR)—which tracks demographic and clinical variables of all Canadian chronic dialysis patients, including variables to assess, such as anemia management, diabetes management, and prevalent fistula rates [8]. Clinical outcomes of these variables were reviewed by the EC-CSN, and areas where targets were not achieved were identified as unperceived learning needs.

Various national nephrology societies (e.g., the CSN, the National Kidney Foundation (NKF)) publish guidelines for management of patients with kidney disease, on dialysis or otherwise. Medical experts from these societies systematically review the literature, rank the level of evidence, and if there is no evidence, they form an opinion statement by consensus. The guidelines are then rated on the strength to which they are recommended; for an example, see the KDOQI Clinical Practice Guideline for Hemodialysis Adequacy: 2015 Update [9]. The societies then disseminate their guidelines via their websites, the published literature, and continuing medical education events. While individual nephrologists often disagree with many of the recommendations put forth by these guidelines, knowledge of these guidelines is integral to the practice of nephrology in Canada. As such, failure to achieve targets described in these guidelines may indicate either disagreement with them, or lack of knowledge of these guidelines, and in turn with the subject matter and literature that subsumes them.

For this analysis, data on the following parameters were generated nationally and by center, as of December 31, 2013, in order to determine achievement of published CSN/NKF guideline targets [10]:

1. Prevalent hemodialysis patients using each of fistulae, grafts, and catheters as their form of vascular access
2. Prevalent hemodialysis patients with fistulae/grafts who are monitored via access flow or recirculation techniques
3. Prevalent hemodialysis patients with a sessional Kt/V above 1.2
4. Prevalent peritoneal dialysis (PD) patients with a weekly Kt/V (PD + renal) above 1.7
5. Prevalent dialysis patients with a predialysis hemoglobin concentration of 100–120 g/L
6. Prevalent dialysis patients with a transferrin saturation of 20–50 %
7. Prevalent dialysis patients with a ferritin concentration of 100–800 ng/mL
8. Prevalent dialysis patients with a predialysis corrected calcium concentration of 2.1–2.54 mmol/L
9. Prevalent dialysis patients with a predialysis phosphate concentration of 1.13–1.78 mmol/L
10. Prevalent dialysis patients with a parathyroid hormone concentration of 16.5–33.0 pmol/L
11. Prevalent dialysis patients achieving none of the three targets in mineral metabolism
12. Prevalent diabetic dialysis patients with a HgbA1c less than 7.0 %

Even if knowledge of these guidelines (and agreement with them) among CSN members were perfect, it is not expected that achievement of these guideline targets would be 100 %, because factors other than knowledge deficit or nephrologist agreement could detract from such achievement. Such factors include but are not limited to (1) disease-related factors (e.g., severity); (2) patient-related factors (e.g., non-adherence with therapy); (3) health system-related factors (e.g., cost of therapy, availability of surgeons); and (4) guideline-related factors (e.g., a target range may be very narrow and therefore difficult to sustain). To get around this issue, a panel of experts from the CSN (13 experienced nephrologists from across Canada) were asked their opinions on what an acceptable achievement rate (within each dialysis unit) for each parameter would be, if knowledge/agreement thereof were perfect. In other words, the panel was asked to suggest an acceptable achievement rate (within each dialysis unit) for each guideline target if the factors listed above (disease-, patient-, health system-, and guideline-related) were taken into account. The values suggested by these experts were then pooled, and these values (“acceptable achievement rates”) are shown in Table 1. For example, the panel felt that if less than 31.9 % of hemodialysis patients in a particular unit used central venous catheters, then that unit was considered to have “achieved the guideline target.” This is because there are many reasons that have nothing to do with nephrologists’ knowledge/preferences to explain a high rate of catheter usage—e.g., vascular anatomy, availability of surgeons, patient preference. For another example, the panel felt that if more than 71.4 % of patients in a particular hemodialysis unit had a Kt/V > 1.2, then this unit would be considered to have “achieved the guideline target”. In this fashion, nephrologists working at centers that fell below this suggested achievement rate for a particular guideline could be said to either disagree with the guideline or have a possible knowledge deficit with regard to that guideline. In both cases, a learning need would be suggested. The national rate of center failure to achieve each guideline target was then calculated and ranked from lowest to highest, to get a sense of where the greatest learning needs lied. Finally, those guideline targets that had less than a 33 % achievement rate across Canada were considered “high needs topics,” those guideline targets that had an achievement rate between 33 and 66 % across Canada were considered “intermediate needs topics,” and those guideline targets that had an achievement rate above 66 % across Canada were considered “low needs topics.”

**Table 1:** Acceptable guideline target achievement rates by parameter, within dialysis units

Parameter	Acceptable guideline target achievement rate (%)
Prevalent HD pts using fistulae	>51.0
Prevalent HD pts using catheters	<31.9
Prevalent HD pts monitored via Qa	>68.4
Prevalent HD pts monitored via Recirc	>39.4
Prevalent HD pts not monitored	<15.3
Prevalent HD pts with sessional Kt/V >1.2	>71.4
Prevalent PD pts with weekly Kt/V (PD + renal) >1.7	>66.5
Prevalent dialysis pts; [Hgb] 100–120 g/L	>57.5
Prevalent dialysis pts; tsat 20–50 %	>54.8
Prevalent dialysis pts; [ferritin] 100–800 ng/mL	>58.0
Prevalent dialysis pts; [Ca] 2.1–2.54 mmol/L	>58.8
Prevalent dialysis pts; [PO4] 1.13–1.78 mmol/L	>52.0
Prevalent dialysis pts; [PTH] 16.5–33.0 pmol/L	>51.7
Prevalent dialysis pts; 0 in target	<15.5
Prevalent dialysis pts; HgbA1c <7.0 %	>44.7

*Abbreviations*: *HD pts* hemodialysis patients, *PD pts* peritoneal dialysis patients, *Qa* access flow technique, *Recirc* recirculation measurement, *[Hgb]* predialysis hemoglobin concentration, *tsat* transferrin saturation, *[ferritin]* ferritin concentration, *[Ca]* predialysis corrected calcium concentration, *[PO4]* predialysis phosphate concentration, *[PTH]* parathormone concentration, *0 in target* percentage of patients with zero mineral metabolism parameters ([Ca], [PO4], [PTH]) within the target range

(C) Comparison of perceived and unperceived needs assessments

The validity of a needs assessment is improved by using multiple methods to assess for these needs and then by comparing the results obtained by each method. This process is referred to as “triangulation.” A number of chronic dialysis-related topics were assessable by multiple methods—questionnaire and achievement of guideline targets; these topics included vascular access type, vascular access monitoring, adequacy, anemia, mineral metabolism, and metabolic issues (e.g., diabetes control). Based on the methods described in sections A and B above, each of the chronic dialysis-related topics was rated as either “high needs,” “intermediate needs,” or “low needs” for each of the perceived and unperceived needs assessments. The results of the two assessments were then compared, qualitatively, in order to define the overall need.

## Results

(A) Perceived needs assessment

The results of the survey are shown in Tables 2 and 3. Altogether, 171 (16.0 %) potential conference attendees responded. Overall, the CSN membership was most interested in the general nephrology topics of “Primary and Secondary Glomerulonephritis,” “Transplant Issues for the General Nephrologist,” “General CKD Prevention – proteinuria, lipids, BP,” “Fluids/Electrolytes/Acid-Base,” and “Geriatric Nephrology.” As for chronic dialysis topics, the membership was most interested in “Volume Status and Hypertension in Dialysis,” “Dialysis Access – Infectious Complications (HD and PD),” “Dialysis Program Administration,” “Cardiovascular Disease in CKD and Dialysis,” and “Hemodialysis Access – Timing, Selection, Surveillance, Intervention.” There was limited interest in “General CKD Staging – Scr, Ccr, Cockcroft-Gault, eGFR,” “Metabolic Issues in CKD and Dialysis – lipids, diabetes management,” and “Psychosocial Issues and Palliative Care in Nephrology.” The topics listed as “Other” were write-in topics; as such, the number of votes for each is expected to be low.

**Table 2:** Perceived needs assessment for the 2016 CSN AGM

Rank	Topic	Votes	% of all votes
1	Volume Status and Hypertension in Dialysis	40	23.4
2	Dialysis Access – Infectious Complications (HD and PD)	31	18.0
3	Dialysis Program Administration	30	17.5
4	Cardiovascular Disease in CKD and Dialysis	27	15.8
4	Hemodialysis Access – timing, selection, surveillance, intervention	27	15.8
6	Outcomes in Dialysis including Adequacy and Timing	24	14.0
7	Dialysis Modality Selection	21	12.3
8	Nocturnal/Quotidian Dialysis	20	11.7
9	Exercise/Rehab in CKD and Dialysis	18	10.5
10	Anemia Management in CKD and Dialysis	17	9.9
10	Mineral Metabolism in CKD and Dialysis	17	9.9
12	Nutrition in CKD and Dialysis	15	8.8
13	Metabolic Issues in CKD and Dialysis – lipids, diabetes	12	7.0
14	Psychosocial Issues and Palliative Care in Nephrology	11	6.4
15	OTHER: “PD”	4	2.3
16	OTHER: Acute Dialysis Modality Selection	2	1.8

Chronic dialysis topics

**Table 3:** Perceived needs assessment for the 2016 CSN AGM

Rank	Topic	Votes	% of all votes
1	Secondary GN	40	23.4
2	General CKD prevention – proteinuria, lipids, BP	37	21.6
3	Primary GN	35	20.4
4	Transplant Issues for the General Nephrologist	33	19.3
5	Fluids/Electrolytes/Acid-base	32	18.7
6	Geriatric Nephrology	31	18.0
7	Ethics in Nephrology	29	17.0
8	Pregnancy and Renal Disease	28	16.4
9	Tubulointerstitial Nephritis	21	12.3
10	Ischemic Nephropathy and Renovascular Hypertension	20	11.7
11	ARF/AKI – Staging and Therapy	19	11.1
12	Diabetic Nephropathy	18	10.5
13	Essential Hypertension	17	9.9
13	Genetic Diseases including PCKD	17	9.9
13	Radiographic Interventions and the Kidney	17	9.9
16	Thrombotic Microangiopathy	16	9.4
16	Nephrolithiasis	16	9.4
16	Management of Overdoses	16	9.4
19	General CKD Staging – Scr, Ccr, Cockcroft-Gault, eGFR	12	7.0
20	OTHER: CAKUT	4	2.3
20	OTHER: CKD Screening	4	2.3
20	OTHER: Renal Pathology	4	2.3
23	OTHER: Onconephrology	1	0.6
23	OTHER: CQI	1	0.6

General nephrology topics

(B) Unperceived needs assessment

The results of this assessment are shown in Table 4, which shows the overall achievement rates nationally, by center, with the various guideline targets. The second column reports the number of centers that submitted data regarding the particular parameter. To arrive at the data in the third column, CORR first reported the percentage of patients for each center that fell within the desired target range. The CSN expert panel then determined what an acceptable achievement rate for each guideline target (as relates to each parameter) for each center should be, assuming that knowledge/agreement of said guideline within the center were perfect. Column 3 therefore represents the number of centers that met this target. The fourth column is the ratio of column 3 to column 2 and therefore represents the percentage of centers considered to have achieved each guideline target at an acceptable rate.

**Table 4:** Unperceived needs assessment for the 2016 CSN AGM—achievement rates for various guidelines targets

Parameter	Centers submitting data	Centers with an acceptable guideline target achievement rate	Percentage of centers with an acceptable guideline target achievement rate
1. HD access			
AVF rate	76	23	30.2
Catheter rate	76	6	7.9
**Total access rate**	152	29	**19.1**
2. Access monitoring			
AVF—Qa	76	46	60.5
AVF—Recirc	76	24	31.6
AVF—NM	76	49	64.5
AVG—Qa	57	40	70.2
AVG—Recirc	57	19	33.3
AVG—NM	57	46	80.1
**Total monitoring**	399	224	**56.1**
3. Adequacy			
HD	75	36	48.0
PD	38	36	94.7
**Total adequacy**	113	72	**63.7**
4. Anemia			
[Hgb]—HD	76	56	73.7
Tsat—HD	76	70	92.1
[ferritin]—HD	74	55	74.3
[Hgb]—PD	51	22	43.1
Tsat—PD	50	48	96.0
[ferritin]—PD	50	46	92.0
**Total anemia**	377	297	**78.8**
5. MM			
[Ca]—HD	76	69	90.8
[PO4]—HD	76	19	25.0
[PTH]—HD	76	0	0.0
0 in target—HD	76	55	72.4
[Ca]—PD	51	41	80.4
[PO4]—PD	51	29	56.9
[PTH]—PD	50	1	2.0
0 in target—PD	50	39	78.0
**Total MM**	506	253	**50.0**
6. DM			
HgbA1c—HD	75	66	88.0
HgbA1c—PD	51	31	60.8
**Total DM**	126	97	**77.0**

*Abbreviations*: *HD* hemodialysis, *PD* peritoneal dialysis, *AVF* arteriovenous fistula, *AVG* arteriovenous graft, *Qa* access flow technique, *Recirc* recirculation measurement, *NM* not monitored, *[Hgb]* predialysis hemoglobin concentration, *Tsat* transferrin saturation, *MM* mineral metabolism, *[Ca]* predialyis corrected calcium concentration, *[PO4]* predialysis phosphate concentration, *[PTH]* parathormone concentration, *0 in target* number of centers with zero mineral metabolism parameters within the target range, *DM* diabetes mellitus

As can be seen from the first column, there are six broad areas where data and guidelines are available—hemodialysis access, access monitoring, adequacy, anemia, mineral metabolism, and diabetes mellitus. Within each broad area, there are between two and eight individual parameters for which data are available. Within each of these six broad areas, the totals for each parameter are summed, and an overall achievement rate for each broad area has been calculated—shown in boldface.

Based on these overall achievement rates for each broad area, it can be concluded that the greatest potential knowledge deficit, and therefore unperceived learning need, is in the area of vascular access creation. Mineral metabolism comes in second place, followed by access monitoring. The broad areas with the highest rates of guideline target achievement and therefore the lowest unperceived learning needs are in the areas of anemia and diabetes mellitus management.

(C) Triangulation—comparison of perceived and unperceived needs assessments

Table 5 shows the results of this triangulation. Each topic was classified qualitatively as being a “high needs topic,” an “intermediate needs topic,” or a “low needs topic,” for each technique that assessed it. From this table, it can be seen that, for the dialysis-related topics, “Vascular Access Type” and “Vascular Access Monitoring” were determined to be areas with the highest learning needs, and “Metabolic Issues (DM)” and “Anemia Management” were determined to be topics with the lowest learning needs.

**Table 5:** Triangulation of needs assessment for dialysis topics

	Survey	Achievement of guideline targets
Vascular access type	High needs	High needs
Access monitoring	High needs	Intermediate needs
Adequacy	Intermediate needs	Intermediate needs
Mineral metabolism	Low needs	Intermediate needs
Metabolic issues (DM)	Low needs	Low needs
Anemia management	Low needs	Low needs

## Discussion

The EC-CSN is in the process of using the results of the above described needs assessment in the planning of the 2016 CSN AGM. As mentioned, the issues with the highest identified perceived and/or unperceived learning needs include topics related to glomerulonephritis and to vascular access on dialysis.

To our knowledge, this is the first published example of a needs assessment for a nephrology CME event that considers both perceived and unperceived needs. That being said, there are numerous published examples of needs assessments for CME. Most of these are perceived needs assessments that utilize survey questionnaires, including Curran et al. in 2007 (family medicine CME in Canada) [11], Turner et al. in 2006 (multiple sclerosis in the USA) [12], Turner et al. in 2004 (occupational medicine) [13], and Purdy in 2002 (migraine) [14]. Dupuis et al. conducted a perceived needs assessment related to Crohn’s disease that utilized a triangulated, mixed-method approach—questionnaire plus semi-structured interview [15].

There are also examples of unperceived needs assessments for CME: Aeschilmann et al. utilized a multiple choice quiz to assess learning needs for rheumatology CME [16]. Klein et al. utilized an environmental scan, in the form of an electronic clinical practice audit, to conduct their needs assessment for family medicine CME in Canada [17]. Finally, Laidlaw et al. conducted a needs assessment of general practitioners for malignant melanoma that utilized multiple methods and considered both perceived and unperceived needs—surveys of general practitioners and specialists (perceived needs), plus literature review and advisory group recommendation review (unperceived needs) [18].

There are a number of accepted techniques for conducting perceived needs assessments for CME. The tool most commonly used is the survey questionnaire, which elicits written responses to multiple questions. Strengths of this technique include efficiency in terms of material and human resources, their ability to address a wide range of topics, and the fact that the information can be returned in a standardized fashion (if, for example, multiple choice questions are used). Conversely, problems can arise when response rates are low (sometimes necessitating expensive follow-up efforts) and if the surveys themselves are poorly constructed [19]. The EC-CSN selected this method for its perceived needs assessment because of these indicated strengths.

Additional published strategies for perceived needs assessments include interviews and focus groups (“group interviews”). These methods acquire personalized, in-depth information; in focus groups, members draw from one another to enhance the information provided. These, however, are very time-consuming and resource-intensive to conduct [5]. For these latter reasons, the EC-CSN did not utilize these techniques.

There are also a number of accepted strategies for conducting unperceived needs assessments for CME. A popular one is the environmental scan. For this technique, existing information/documents are scanned unobtrusively for learning needs. These documents could include minutes of meetings, chart audits, attendance data, national databases, published guidelines of care, and literature searches, among others. A major strength of this technique lies in its economical sources of data, i.e., documents have already been produced for another purpose, and so no new expenditures are required. Further, a wide spectrum of data is available, the data are often generated iteratively, and it can be scanned unobtrusively. On the other hand, weaknesses include data that may be too broadly defined, too time-consuming and costly to analyze, and the fact that there can be political interference with accessing/analyzing the data [5, 20]. The CORR database was deemed an ideal selection for data as it was freely offered for review by its governing board (in a de-politicized manner), and no costs were attached to its review.

Other published strategies for unperceived needs assessments include chart audits and pre-course testing. These provide objective information but can be very time-consuming to conduct [5, 16]. For these reasons, the EC-CSN did not utilize these techniques.

Finally, the published literature suggests that the validity of the needs assessment is increased if multiple techniques are used, and the results of these techniques are compared—a process known as triangulation [5]. Our study utilizes such a technique which we feel strengthens our results in terms of validity.

There are a number of strengths to the present study. It represents the first published study of a needs assessment for a nephrology CME event that considers both perceived and unperceived needs. It therefore demonstrates that such work can be done in order to enhance the delivery of nephrology CME. Second, for the perceived needs assessment, a reasonably representative sample of Canadian nephrologists—171 individuals—responded to the survey, enhancing its validity. Third, for the unperceived needs assessment, a robust database that represents dialysis practice throughout Canada was available for analysis. Fourth, triangulation of the different methods utilized was performed, further enhancing the validity of the work.

We acknowledge that there are some limitations to the study. For the perceived needs assessment, the topics for the survey were determined by the EC-CSN after much discussion; that said, some of the topics were much more general (e.g., “Dialysis Outcomes”), and others were very specific (e.g., “Radiographic Interventions and the Kidney”). This could have led to some topics being selected more than others by the respondents.

Perhaps the greatest limitation of the unperceived needs assessment is the lack of a standard, validated, published approach for conducting it. The environmental scan, in a general sense, is a validated method, particularly for qualitative data [5]; however, we could identify no standard approach for handling this kind of data. That said, we feel that the method we devised was reasonable—we believe that the panel of experts was able to assess the factors that detract from perfect guideline target achievement and to determine if those factors were nephrologist-related or not. Second, the results were dependent on the availability of CORR data. CORR does not generate data for the non-dialysis-related topics, so the survey results for these topics could not be triangulated with any known reference. Third, the authors concede that the level of evidence cited within published guidelines can be poor; for example, KDOQI’s minimum acceptable target for urea clearance (a single pool Kt/V of 1.2 for thrice weekly dialysis) reflects grade 1B level evidence [9].

A third possible weakness in the unperceived needs assessment is that the targets set by the panel of experts were chosen because they were what the panel felt to be reasonable but were not correlated with patient outcomes. Indeed, the literature does include a number of studies that look at the extent to which achievement of guideline targets predicts outcome. Djukanovic et al. demonstrated that the failure to achieve KDOQI targets for Kt/V, hemoglobin, and PTH was associated with an increased relative risk of time to death in a Serbian hemodialysis population [21]. Anton-Perez et al. demonstrated an inability to meet KDOQI vascular access targets in a Spanish population and showed that this was independently associated with high mortality [22]. On the other hand, Tangri et al. not only demonstrated variable guideline target achievement for various mineral metabolism factors (calcium, phosphate, PTH) in a British renal registry (hemodialysis plus peritoneal dialysis) but also showed that those patients who achieved guideline targets did not have a survival advantage [23]. As such, in our view, there is insufficient evidence in the literature to support the use of outcome-based targets for our needs assessment—not all guidelines are addressed, and those that are addressed arrive at inconsistent conclusions.

For part C, triangulation, as for the unperceived needs assessment, there is no validated, published approach for triangulating this kind of data. Most of the published literature that describes triangulation methods for needs assessments involves qualitative data and utilizes analysis of themes derived from such data sources as interviews and focus groups [15, 18]. We do feel, however, that our method of comparing the rank order of the various topics identified by the two assessment methods is reasonable. Second, the triangulation was performed on data that was not quite contemporaneous: The unperceived needs assessment was conducted on data obtained as of December 2013; the survey questionnaire (perceived needs) was administered in April 2015. This was done in order to use the most current data possible when planning the meeting. The likelihood that this introduced significant error is low because (a) the datasets differ only by 16 months and (b) the results of the unperceived needs assessment have not changed very much over the 5 years that this assessment has been conducted.

## Conclusion

In conclusion, this paper demonstrates that a multi-method needs assessment strategy, that addresses both perceived and unperceived needs, can be generated and applied, so as to enhance the quality of nephrology CME in Canada.
